# Leadership of school principals for school health implementation among primary schools in Mataram, Indonesia: a qualitative study

**DOI:** 10.1186/s41182-023-00568-y

**Published:** 2024-01-02

**Authors:** Hirono Sasaki, Dian Puspita Sari, Cut Warnaini, Fahrin Ramadan Andiwijaya, Rie Takeuchi, Hamsu Kadriyan, Fumiko Shibuya, Jun Kobayashi

**Affiliations:** 1 Department of Global Health, Graduate School of Health Sciences, University of the Ryukyus, 207 Uehara, Nakagami-Gun, Nishihara, Okinawa 903-0215 Japan; 2 Japanese Consortium for Global School Health Research, Okinawa, 207 Uehara, Nakagami-Gun, Nishihara, Okinawa 903-0215 Japan; 3 Faculty of Medicine, University of Mataram, Jalan Pendidikan 37, Mataram, West Nusa Tenggara 83125 Indonesia; 4 Faculty of Medicine, International University of Health and Welfare, 4-3 Kotsunomori, Narita, Chiba 286-8686 Japan

**Keywords:** School health, Primary school, Principal, Leadership, Health-promoting schools, Qualitative study

## Abstract

**Background:**

Health-promoting schools (HPS) are acknowledged as a comprehensive approach to improving children’s health and educational outcomes through learning and school life. Principals are key players in HPS implementation. However, concrete leadership practices in HPS in low- to middle-income countries have not been clarified. Therefore, this study aimed to explore and generate themes surrounding the leadership practices of principals in implementation of school health in Indonesia, a predominantly Muslim country consisting of diverse religions that have expanded HPS at the national level.

**Methods:**

In-depth interviews and focus group discussions (FGDs) were conducted with the principals, teachers, parent representatives, and school board committee members in 10 target schools. FGDs were conducted with school health supervisory board members in Mataram City. All interviews were recorded and transcribed. Thematic analysis was undertaken to generate themes.

**Results:**

The principals demonstrated leadership practices based on their religious beliefs, values, and morals. This may suggest that beliefs and morals support an understanding of their responsibility to ensure the well-being of all school community members, regardless of religion, in a diverse environment that is predominantly Muslim but also multicultural and multi-religious. Further, these beliefs and morals might reinforce implementation of school health. Importantly, the principals’ coordination skills in cooperating with multiple sectors might contribute to successful implementation of school health. Also, principals emphasized they were tasked to develop capacity for implementation of school health. Thus, principals should understand leadership in the implementation of school health as their duty from the training stage to encourage health at the school level.

**Conclusion:**

In this study, “have professional educators’ beliefs and religious beliefs and Indonesia’s morals” was generated as a new theme, whereas several common themes were found as in previous studies. The results of this study suggested the importance of leadership by principals in the implementation of school health. Strengthening the capacity of school principals by integrating the contents of school health leadership practice into pre- and in-service training through the development of a policy on principals’ duties in school health might contribute to the successful implementation of school health.

## Background

The school has been one of the strategic settings for promoting healthy lifestyles to children, adolescents, parents, and communities. Schools are used to disseminate messages focused on health services and health education for specific diseases. However, in 1995, the World Health Organization (WHO) proposed the health-promoting school (HPS), which is a whole-school approach in the Global School Health Initiative. HPS is a concept to enhance children’s health and educational outcomes through learning and school life [1–3]. It consists of six components: (1) healthy school policies, (2) physical environment, (3) social environment, (4) health skills and education, (5) parent and community partnerships, and (6) access to health services [1]. Currently, the HPS concept is being adopted worldwide with a gradual spread to Europe, the North and Latin America, and Southeast Asia [4]. The spread of HPS in Southeast Asia was triggered by the Hashimoto Initiative in 1998, which set out to combat parasites [5]. School-based infectious disease control became an essential foundation for school health in Southeast Asian countries and subsequently led to the development of school health policies in each country [5].

With the expansion of HPS practice, factors involved in the effective implementation of school health were explored. An HPS case study by the Association of South East Asian Nations countries conducted in 2015 suggested that factors for effective implementation of HPS include the establishment of national policies and frameworks for health and education, strong leadership at the national, local, and school levels, and adequate funding and human resources [6]. In addition, the guidelines, which were developed based on effective factors of practice in low- and middle-income countries (LMICs) for the purpose of sustainable implementation of school health by WHO and the United Nations Educational, Scientific, and Cultural Organization (UNESCO), introduced global standards with eight components: (1) government policies and resources, (2) school policies and resources, (3) school governance and leadership, (4) school and community partnership, (5) school curriculum, (6) school social-emotional environment (7) school physical environment, and (8) school health services. In relation to (3) and (4) of these components, the need to define a model of school leadership and governance involving students, schools, community, and national and local government representatives to ensure sustainability of the HPS and school leadership has continued to be one of the effective factors in the implementation of school health [7, 8].

In a school-level intervention for school health, the support and leadership of principals is crucial. According to a previous study in Canada, principals (1) “prime for cultural change”, (2) “advocate for cultural change”, (3) “negotiate, motivate & collaborate”, (4) “monitor & enable others”, and (5) “support & sustain”. The principal is also a key person in integrating HPS into the school culture, establishing the social environment within the school, and maintaining the HPS culture and recommended the application of a shared leadership style [9, 10]. In addition, a study on common features of the successful implementation of school health in Sweden showed that successful leaders synchronized actions by respecting teachers’ work and building the capacity of teachers [10]. However, leadership practices vary depending on the context, such as local history, culture, regional characteristics, and school policy perspectives, in which it exists and require leadership study in a variety of settings [11–13]. Therefore, leadership in the implementation of school health may also need to consider the context in which it exists and the study of school leadership on the implementation of school health in various backgrounds [14, 15]. However, these leadership studies on implementation of school health have been conducted in the context of high-income countries. The authors have found no papers in international journals in English on school leadership for implementation of school health that especially focus on the leadership role of principals in implementing school health in LMICs in Asia.

Indonesia, one of the LMICs that has implemented HPS at the national level, is made up of multi-ethnic and multi-religious people with a Muslim majority. The education system in Indonesia consists of schools under the jurisdiction of the Ministry of Education and Islamic schools under the jurisdiction of the Ministry of Religious Affairs. Compulsory education lasts for 9 years from the age of 7 to 16, which is the age of enrollment in primary and secondary schools [16]. Indonesia’s school health system, Usaha Kesehatan Sekolah (UKS), was introduced in 1959 as a school health project. Since then, the UKS has integrated the HPS concept as part of a comprehensive approach that includes health education, school environment development, and health service delivery and has stipulated that it must be implemented in all primary, middle, and high schools. The UKS, like the HPS, is a comprehensive approach based on health education and health services, but it is unique in that it addresses religion and mental health as important provision. The UKS sets goals in health education for each primary, middle, and high school to teach the fundamentals of a healthy lifestyle through practice and specific examples in primary schools [17]. In Indonesia, approximately 4.6 million children attend primary school, and the net enrollment rate is 93.5% [18]. Thus, HPS implementation for primary school students provides a great opportunity for them to acquire the foundation for a healthy lifestyle in the future. A strong commitment by the central government has led to recommendations to state governors to implement the UKS. The HPS implementation report by UNESCO in 2019 stated that challenges to HPS implementation in Indonesia included the lack of training on UKS and inadequate monitoring and evaluation of UKS implementation. The challenges to HPS practice are tackled with limited human and financial resources [8]. However, in addition to resource issues, our research group observed that the structure of each school regarding the implementation of the UKS is also a challenge during carrying out the previous study related to school health in Indonesia. Although the assignment of a UKS coordinator is actually recommended, it can be assumed that coordinators actually have few opportunities to receive training and lack confidence in the actual implementation of the activity. Therefore, it was considered possible that in practice, many schools where the leadership of the principal is also involved in the UKS may have led to the implementation of the UKS. Previous studies on leadership in Indonesia are mainly related to the education field, such as principal leadership associated with student achievement. It is also suggested that the difficulty of leadership differs between urban and rural areas. Poverty, low awareness of education, low wages, and inadequate governmental support for teacher training are some of the reasons why it is not easy for principals to perform leadership duties [19]. There is no study on leadership focusing on HPS implementation in Indonesia.

Thus, this study aimed to explore practices of primary school principals in promoting HPS and generate a theory of how leadership works, which might contribute to the promotion of HPS and help the school community in Mataram City, Indonesia, to acquire healthy lifestyles. In addition, this study provided findings on the type of leadership required of principals in the further promotion of HPS in LMICs and showed the nature of leadership in Muslim and multi-religious contexts.

## Methods

### Study design

This was a qualitative study, which is an effective way of understanding a phenomenon that is not yet well understood [20]. We collected a variety of qualitative data through in-depth interviews (IDIs) and focus group discussions (FGDs).

### Study site

This study was conducted in Mataram City, West Nusa Tenggara, Indonesia. It is the capital city of West Nusa Tenggara Province and has a diverse religious context consisting of practitioners of Islam (82.4%), Hinduism (14.2%), Christianity (1.6%), Buddhism (1.0%), and Catholicism (0.8%) [21]. We selected Mataram City, which is less affected by economic poverty and educational prevalence [19], as a site to explore leadership practice in a diverse setting with multiple religions.

### Study participants

We included participants from primary schools and school health advisory boards in Mataram City. We recruited 10 primary schools from the participant list of a school health competition (Lomba Sekolah/Madrasah Sehat) in 2019 and 2021 from 150 primary schools in Mataram City because we assumed that these 10 schools implemented UKS to the “optimal” level, as defined by the local government in Indonesia. We targeted school principals and members of school health consisting of UKS coordinators, teacher representatives, parental representatives, and school board committee members for interviews in schools. A school health supervisory committee was established to strengthen the implementation of school health through planning, mentoring, and monitoring by school board members [22]. It consists of members belonging to the following departments: health, education, environment, religious affairs, agriculture, and municipal housing [22]. We invited representatives from the health and education departments, which mainly cooperate with schools in the implementation of UKS [23].

### Data collection and instruments

The data collection was conducted through IDIs and FGDs with co-researchers well experienced with qualitative studies at the site. Data collection instruments for IDIs and FGDs were developed based on objectives referred to in previous studies on implementation of school health [9, 10]. Three types of instruments for IDIs and FGDs were developed: IDIs for school principals, FGDs for school health committee members, and FGDs for school health supervisory board members. Questions on the instruments included (1) the process of implementing the school health activities implemented; (2) the role of the principal in the implementation of school health; and (3) thoughts on leadership for good school health practice.

All IDIs and FGDs were conducted in the Indonesian language. IDIs were carried out in 10 primary schools. The first author and co-researchers visited each school and conducted the IDIs in person, which took from 30 to 60 min. FGDs were conducted for school committee members and school health supervisory board members. FGDs at schools used two methods to carry out infection prevention measures for COVID-19. We conducted onsite FGDs in schools where sufficient physical distance for discussion was available, whereas in schools where sufficient physical distance was not available, FGDs were conducted online by referring to online data collection [24]. We conducted the FGDs for the school health supervisory board at the provincial health office, and these FGDs took from 60 to 90 min. All interviews were conducted using interview instruments. They were recorded with integrated circuit recorders or an application for capturing audio records of online meetings depending on whether the interviews were done on site or as online interviews and transcribed in the Indonesian language. Transcriptions were performed using Google Docs and audio records to ensure the validity and reduce the time for transcribing [24]. The transcriptions were translated from Indonesian to English by a native Indonesian speaker after they were transcribed. Data collection was conducted for 2 months from July to August in 2022.

### Data processing and analysis

Data analysis was conducted using thematic analysis. Thematic analysis allows for flexible extraction of features and themes from data obtained from subjects with different characteristics, and the analysis focused on the subjective world of each case [25, 26]. As a process of analysis, we used a hybrid method in which the raw data itself was transcribed inductively and then interpreted through either the theoretical frame of reference or the hypothesis developed by the authors [27, 28].

Each sentence of the transcripts obtained from the interviews at each school and from the school health advisory board members were coded, and selected codes focusing on “school leadership practices on implementation of school health from the perspective of the principals themselves, teachers, parents, school board members and school health advisory team members” were extracted. These codes were created deductively, referring to the categories generated by the study in Canada that categorized leadership in the implementation of school health [9]. However, for those codes that did not fit into any of the theme in the previous literature, new theme was generated inductively. As described above, themes are generated by a hybrid of deduction and induction, six themes were generated.

We conducted triangulation and member checking to enhance the trustworthiness of the data analysis. At least three researchers, including a local Indonesian researcher, were involved in all steps of the analysis. We examined interviews with different groups such as teachers, parents, school board members, and stakeholders for data triangulation. In addition, the authors were confirmed by co-researchers in Indonesia who are experienced in qualitative research into the steps of creating themes.

## Results

Tables 1 and 2 show the characteristics of the participants. In total, 10 principals from primary schools participated in the IDIs. Their average age was 54.7 years, and their average length of service as a principal was 7.91 years, with 70% of the principals having more than 10 years of experience in conducting school health activities. For the primary school FGDs, 40 participants from 10 primary schools participated, which comprised 27 teachers, 7 parent representatives, and 6 school committee members. For the FGDs with the school health supervisory committee, which was composed of supervisors from both the health and education sectors, 2 participants from the health department’s school health supervisory committee participated, but we could not enroll members of the education department due to scheduling conflicts.

**Table 1:** Characteristics of the principals

Gender	Age (yrs)	Length of service as a teacher	Length of service as a principal	Length of service in the current school	Course history of training	Experience with implementation of school health
Female	57	36 years	11 years	5 months	Strengthening of leadership roles	11 years
Male	59	39 years	16 years	1 year	Strengthening of leadership roles	39 years
Male	51	17 years	2 months	2 months	–	5 years
Female	55	24 years	6 years	4 years	Training for principal candidate	10 years
Female	49	24 years	5 years	5 years	Training for principal candidate	8 years
Female	60	38 years	5.5 years	5.5 years	Training for principal candidate	4 years
Male	59	39 years	16 years	1 year	Strengthening of leadership roles	39 years
Female	47	19 years	2 years	2 years	Training for principal candidate	10 years
Female	52	31 years	4.6 months	6 months	Training for principal candidate	15 years
Female	58	27 years	24 years	24 years	Management workshop	24 years

**Table 2:** Characteristics of participants in the focus group discussions (*N* = 40)

Characteristic	*n*
Teachers (*n* = 27)	
Male	5
Female	22
Age (years), average	39.6
Length of service as a teacher (years)	
< 10	3
10–19	15
20–30	6
> 30	3
In current school (years)	
< 5	5
5–10	6
> 10	14
No answer	2
Parents’ representatives (*n* = 7)	
Male	1
Female	6
School board committee members (*n* = 6)	
Male	5
Female	1
School health supervisory board (*n* = 2)	
Male	0
Female	2

As shown in Table 3, six themes comprising 19 categories were generated. The themes were (i) have professional educators’ beliefs and religious beliefs and Indonesia’s morals; (ii) share a vision for cultural change; (iii) build and maintain a foundation to require UKS guideline; (iv) support implementation of school health by capacity building and work organization, (v) advocate school health’s value to in and out of school; and (vi) sustain and support by motivating and reminding others. The six themes and the categories supporting these themes are detailed below.

**Table 3:** Themes and categories related to leadership in school principal

Themes (6)	Categories (19)
Have professional educators’ beliefs and religious beliefs and Indonesia’s morals	Religious beliefs of the principal
	Values and morals as a professional educator
Share a vision for the cultural change	Understanding of school context
	Set school health activity as a school priority
	Role model
	Created regular school health activities
	Build a good relationship within school
Build and maintain a foundation to require UKS guideline	Assign teachers to take responsibility
	Set up physical environments
	Secure the budget from various resources
	Cooperation with multiple sectors
Support school health implementation by capacity building and work organization	Monitor implementation of school health activities
	Enable teachers and children to take ownership
	Provide an opportunity to acquire skill and knowledge
Advocate school health’s value to in and out of school	Communicate the importance of school health with school community
	Act as a spokesperson to the school community
Sustain and support by motivating and reminding others	Reward, recognize, and celebrate
	Work as a partner with teachers and the community
	Keep making small changes

### (i) Have professional educators’ beliefs and religious beliefs and Indonesia’s morals

It was suggested that the principals’ leadership be based on their religious beliefs and values as a principal, and that they practice leadership in accordance with those beliefs and values. In the statements of the principals of three of the ten schools, it was confirmed that their religious beliefs, in addition to their beliefs as educational professionals, were linked to the exercise of their leadership.

#### Religious beliefs of the principal

The principals spoke of “Amanah”, the value of having a responsibility to one’s superiors, colleagues, and other school members. It was a value according to which the principals considered their job as something entrusted to them by God.

*“…we all are partners, we all work together. This is Amanah (something entrusted to me) and responsibility to the Government and God. …” (Primary School_IDIs_1)*

In addition, religious beliefs reflect health values, and this became one of the reasons to apply school health at school.

*“It’s because health is our needs. Even if it’s not for competition, why not we do it? Maintaining good health is our duty, right? Good health is part of our faith (for Muslim people).” (Primary School _IDIs_1)*

#### Values and morals as a professional educator

In contrast, some principals considered not only religious values in implementing school health, but also values as educators responsible for the healthy development of their students.

*“…puberty education for children, I think that’s an important thing and not a taboo thing. They have to get the right information, at least we’re decreasing future risk because they understand more about puberty.” (Primary School_IDIs_10)*

### (ii) Share a vision for the cultural change

The school principals took the initiative in cultural change within their schools by sharing visions based on the understanding of the situation at their individual schools.

#### Understanding of school context

In preparation for the implementation of school health, the principals formulated a vision by understanding the school context.

*“At the beginning of the school year there was a meeting with the principal, we were also present. At the meeting, the principal wanted to ask about all the parts. Say UKS, or some other part. Ask each program. What program is needed, which is considered a priority, is usually the school principal, who will immediately look for the budget. So she immediately took notes so that there were no obstacles to implementing what was proposed by the teachers at the school.”(Primary school_FGDs_3_5)*

#### Set school health activity as a school priority

The setting of implementation of school health as a school priority through sharing the vision, including plans, goals, and mottos, in staff meetings.

*“I’ll make a motto first, our motto here is to be in tune with our steps towards success. So, every step we have to be in rhythm because if there is one that is not there then we will get failure.” (Primary School_IDIs_4)*

#### Role model

In addition, principals encouraged compliance by serving as good examples. For example, one principal took the initiative to stand at the school gate to make sure that students’ clothes, hair, and nails were clean and hygienic, that the restrooms and classrooms were clean, and that trash was picked up from the school grounds.

*“Giving an actual living example. The example of life means doing what I said. Dressing neatly, even though it is not dashing, but it does show that we are clean. So, an example important. Showing a good example to the children and teacher both in everyday life. “(Primary School_IDIs_3)*

#### Create regular school health activities

The school principals integrated this initiative into the school’s daily routine. Specifically, principals allocated time during staff meetings to communicate the importance of this initiative. Additionally, extracurricular activities also were integrated into the school’s schedule. For instance, some principals implemented school health activities by designating certain days of the week for morning physical activities or educational breakfast sessions.

*“So we took Thursday to conduct the “Breakfast Together” and every teacher in each class supervised the students’ food. Were the food already healthy and we always remind the students to not bring any junk food.” (Primary School_FGDs_5_1)*

#### Build a good relationship within school

Also, principals established a collaborative relationship within the school for the implementation of school health. One principal highlighted transparency as a crucial aspect in building trust and a collaborative environment within the school.

*“The first is cooperation, mutual cooperation, and transparency. This is the most important. So, there are no lies between us, and any problems that we can immediately inform, so that friends are not uninformed. We get together almost every day for a small meeting every time the kids get home from school. The togetherness we built.” (Primary school_IDIs_3)*

### (iii) Build and maintain a foundation to require UKS guideline

In this theme, principals established a foundation for implementation of school health by creating a physical environment, managing the budget, and working with multiple sectors.

#### Assign teachers to take responsibility

All principals appointed a teacher to be in charge of school health based on the UKS guideline and shared responsibility for the implementation of school health activities.

*“The principal program given to us teachers, is according to our respective portions. Like me, I am a sports teacher, and I was given the responsibility of being a UKS teacher and executed to the best of our ability. We are ready to do it while we can.” (Primary school_FGDs_3_4)*

#### Set up physical environments

The principals also undertook improvement of the physical environment according to the standards set forth in the UKS.

*“It is our job together in this school. We take care of everything about cleanliness. For the student, there are many schools health that the teacher still. For example, playing bell they passed through the yard and picked up the trash and put the trash away without a guide anymore. So, it’s normal to keep it clean and keep schoolyard clean.” (Primary School _IDIs_3)*

#### Secure the budget from various resources

Some principals were described as being able to manage the school’s financial resources and seek out other sources of school funding. They utilized a significant portion of the financial resources for implementation of school health that was procured from the Bantuan Operasional Sekolah (BOS). BOS is the School Operational Fund that was introduced in 2005 by the Government of Indonesia and provides support for schools and madrasahs offering basic education through funding for non-personnel operational budgets. The program, which was established as part of the government’s compulsory basic education initiative, specifically focuses on providing financial resources for the day-to-day operations of these institutions [29]. Additionally, some principals solicited material support from parents and school board committees to be used in school health programs.

“For assistance from the parents, it seems we have just assisted the gardening of the school. To beautify the school, that’s all. We help like buying flowers, plants, to beautify the school. That’s all from the parents” (Primary school_FGDs_1_4)

#### Cooperation with multiple sectors

The principals stated that multi-sector collaboration could not be removed in creating this foundation. The school principals also used social networking sites to receive training and services information related to school health management from the public sector, non-governmental organizations sector, and the private sector.

“There are several agencies that are indeed collaborating with schools, namely Puskesmas (Pusat Kesehatan Masyarakat) (Public Health Center). Puskesmas takes a pretty good role.” (Primary School _IDIs_2)

### (iv) Support school health implementation by capacity building and work organization

The principals organized the school structure to enable the conduction of monitoring and evaluation and to set up training opportunities to enhance the knowledge and practical skills of the teachers and students.

#### Monitor implementation of school health activities

At this point, the principals were open to listening to problems and criticism from teachers and the community while clarifying the overall direction of implementation. The principals then worked with teachers to prioritize how to incorporate this project through monitoring and evaluation of the implementation.

*“Then the principal also needs to evaluate because sometimes there are new programs that are unclear during implementation, because the process is confusing (for the implementer) …. now that is what we do so that when we tell the problem and so on, we can discuss together…” (Primary School_FGDs_3_1)*

#### Enable teachers and children to take ownership

To make this possible, the principals also considered restructuring the school health team within the school to enable teachers to take the initiative in problem solving.

*“Here, the UKS coordinator, the canteen coordinator, the religious coordinator, and the waste selection coordinator are divided. We have shared all of this, and they are the ones who will work.” (Primary School_IDIs_4)*

#### Provide an opportunity to acquire skills and knowledge

Also, the principals created opportunities to develop teacher and student capacities for implementation of school health.

*“…from the training we have received from Plan International, our children are also taught to become sanitation ambassadors where they will develop and teach their friends at school how to keep the bathroom clean, especially those related to MKM (menstrual health management).” (Primary school_IDIs_3)*

### (v) Advocate school health’s value to in and out of school

The principals acted as advocates of the value and importance of school health to the school community while involving and encouraging teachers and students to participate in school health.

#### Communicate the importance of school health

Principals worked to ensure that teachers, parents, and students understood the value and importance of a healthy lifestyle.

*“I only took the initiative to show our school that the good we do have value, so I am grateful that in the end all parties support it because this is not to teach healthy schools first but to teach culture for our school.” (Primary School_IDIs_5)*

#### Act as a spokesperson to the school community

Principals understood their role of engaging with parent groups and serving as communicators to the school community to get them involved in school health activities. They disseminated information regarding school health activities through various channels, including school assemblies, school events, and digital platforms.

*“So, I have called when there is a healthy school competition to call parents to socialize what our goals are so that they are very supportive and want this school to be healthy, but our main goal is not only healthy schools but to differentiate this for children including parents so that parents when littering children are able to reprimand their parents.” (Primary School_IDIs_3)*

### (vi) Sustain and support by motivating and reminding others

In this theme, principals emphasized the importance of reminding and motivating students, teachers, and the community. The importance of undertaking the process of change slowly for it to become embedded within the school was mentioned.

#### Reward, recognize, and celebrate

Principals praised the positive efforts of other teachers and students to celebrate progress in making changes. This is important to ensure the continued involvement of others in the school community.

*“The motivation is when I give a reward to the teacher. So, this is the motivation. In addition, during the ceremony the children also get rewards…If the reward is big, we may not be able to afford it, if we are together, if we have a little money, we may take it for a picnic. So together in joy and sorrow.” (Primary School_IDIs_4)*

#### Work as a partner with teachers and community

In addition, principals emphasized the importance of partnering with teachers and the community. They stated that despite their position as leader, they listen to input and feedback from teachers and the community and encourage collaboration to work together.

*“I really appreciate the teacher, whatever we do must involve all parties. We can’t possibly work alone, if we want a clean school, we have to work together with all the teachers… What needs not involved, it will offend, so that with good cooperation, all of us, residents, school then hopefully nothing is difficult. Because whatever I want to do, gather all parties because we help each other so good cooperation is needed” (Primary school_IDIs_4)*

#### Keep making small changes

Principals emphasized here is that it takes time to change the behavior at a school, so it is important to begin with actions that can be implemented within the school and to continue to work on an ongoing basis.

*“The challenge is changing the behavior. This is so difficult. What implement at school not necessarily applied at home and in community. At least there’s a good collaboration among government, families, and school. Without a good cooperation among these three components, well I think… (it would be difficult) … Let’s start from ourselves, do not need to see others, from small things in our school environment. If we have taught good behaviors at school, we hope those behaviors can also be practiced at home. Let’s focus on our plate first.” (Primary school_IDIs_1)*

## Discussion

This study identified the leadership practices that support the implementation of school health in the context of Mataram City, Indonesia, a developing country with a Muslim majority. While some of the inductively generated themes were similar to those in prior literature, the themes related to beliefs and morals, which is “Have professional educators’ beliefs and religious beliefs and Indonesia’s morals” were considered novel. The other five themes were similar to five following themes which is generated by previous research in Canada: “prime for cultural change”, “advocate for cultural change”, “negotiate, motivate & collaborate”, “monitor & enable others”, and “support & sustain”.

The principals demonstrated leadership practices based on their values, religious beliefs, and morals in their implementation of school health. “Amanah” is one of the unique Islamic values of leadership that maintains the trust of the teacher, students, and community, delegating all matters to be managed for mutual benefit, which is entrusted to the principal by God [30, 31]. This value could give them confidence in their decisions on and implementation of school health as well as for school management [30]. It was also suggested that religious beliefs reflect health values, which may have been another reason for implementation of school health. Religion provides discipline and influences personal well-being [32]. Islamic culture emphasizes the importance of health, such as personal hygiene, stress management, and healthy eating [33]. Thus, the value of health in religious beliefs might have reinforced the practice as a reason for the implementation of school health.

Religious education has an important role in the promotion of both physical and mental health in school [34]. In Indonesia, religion is integrated into general education in combination with moral education to develop students’ character [35]. A previous study stated that character education affects the prevention of bullying, stress management, and related mental health, as well as use of substances such as tobacco and alcohol [34]. In other Islamic countries, Islamic education in general schools is also often limited; however, in Indonesia, other religious education courses, including Islamic education, are mandatory [35]. In addition, not only Islamic education but also other religious education contributes to the promotion of a healthy lifestyle in school. For example, Catholic education has also noted a connection between the health and physical education curriculum and the religious education curriculum [34]. It is stated that promoting personal growth, developing concepts and skills for physical activity, and taking a position on health and physical education to promote individual and community health can all be associated with the religious education curriculum in Catholic education [36].

The principals recognized their role in taking responsibility for ensuring the well-being of all people in the school community in diverse settings. Indonesia is a multi-religious country, and although Islam is the majority religion, other religions are also respected. Pancasila is the foundation principle of Indonesia and part of the national education in Indonesia. It consists of five principles: (i) belief in one God; (ii) just and civilized humanity, including tolerance of all people; (iii) unity of Indonesia; (iv) democracy led by the wisdom of deliberation among representatives of the people; and (v) social justice for all. The curriculum on religious education is reflected in Pancasila [34]. It is divided into six categories: Islam, Protestantism, Catholicism, Hinduism, Buddhism, and Confucianism, and the content is based on the practice of each religion’s beliefs. The goal of this curriculum is character building common to all six religions and is expected to be attained through the practice of each religion [37]. Moreover, Islam originally has a background of tolerating other religions [38]. The principals understood religious diversity, which is described in the national education strategy, respected non-Islamic cultures, and took responsibility to ensure a healthy lifestyle for the entire community through leadership with consideration of diversity.

The principals’ skills in the coordination of multi-sector cooperation might be essential for creating a foundation for and maintaining the implementation of school health. The UKS guideline in Indonesia mentions that each school is required to assign UKS coordinators who coordinate in the implementation of school health for coordinate resources inside and outside the school [19]. However, not all schools have them, and the reality is that principals are responsible for them. In schools, it is necessary to coordinate not only with school nurses and counselors who directly contribute to health services, but also with the homeroom teachers in each class. In addition, school health-related subjects are multi-subject and require coordination among subject supervisors. A collaboration with institutions outside school such as health centers, non-governmental organizations, or private companies is essential for implementing school health activities such as health services, health education, and environmental improvement. Some principals had concluded Memoranda of Understanding to strengthen cooperation with related organizations and enhance school health activities. This is true not only in Indonesia, but also in other LMICs, in which schools tend not to have a clearly defined person in charge of school health [38, 39]. Therefore, the principal is expected to provide leadership as the point of contact for the school and to collaborate and coordinate with multiple sectors.

The principals emphasized that they were actively involved in developing the school organization and teachers’ and children’s abilities for the implementation of school health. Capacity building, which was not addressed as a theme in previous studies, was chosen as the topic of this study because many principals in this study mentioned working as a team to implement school health. In Indonesia, the UKS guidelines recommend the creation of UKS teams to maintain the implementation of school health activities [19]. However, the implementation tends to depend on the discretion of the principal. According to a report from the Java Island district of Indonesia, most of the schools have not organized UKS teams [40]. In this study site, all participating schools selected Physical Education teacher as a UKS coordinator. However, we did not observe that they were making adequate coordination. And they did not have opportunities for training on school health implementation. In Canada, the Education Act at the provincial level clearly states that the role of the principal is to organize and build the capacity of teams and individuals to implement projects [41]. These roles of principals are also used in the implementation of school health. In Indonesia, in addition to the UKS coordinators, the leadership of principals is considered to be a key factor for effective implementation. Principal leadership may also be an important factor in promoting school health, as human resource capacity for school health is generally low in many LMICs.

School principals should understand from their training stage that school health is their duty by taking actions “to encourage organization and capacity building on school health” that is explicit in the policy. The findings of the present study suggest that strengthening capacity building of school principals is a key to the successful promotion of school health. The concept of capacity building originated from the field of development assistance, where it is a concept aimed at developing the necessary prerequisites for the implementation of successful and sustainable health promotion including human resources [42]. A previous study pointed out the need to build an environment, such as human resources development, that needs to be provided on the national and regional levels for health promotion in schools [43]. The development of human resources pre-service training and in-service training is a crucial function [36]. In Japan, a person aspiring to become a principal must have the prescribed teaching experience and must pass an examination [44, 45]. However, the process of principal preparation training is different in each country. The US and Finland, for example, require aspiring principals to have a master’s degree in education whereas other countries require teaching qualifications [42]. In Indonesia, the process to become a principal consists of recruitment, selection, training, and certification [46]. Candidates for principal may participate in the training if they meet the minimum academic standards. After attending this training and achieving at least the minimum passing grade for the training, the principal can finally be certified [43]. However, there is a need to improve and strengthen principal leadership training as it has been reported that the process of principal selection is inappropriate due to the selection being conducted based on political connections rather than performance and skill [47]. The leadership of the principal is important to promote organization and capacity building in the implementation of school health. Thus, a clear statement on the implementation of school health as a responsibility of the principal in the policy and promoting the principal’s understanding of school health from the principal’s training stage could contribute to the implementation of school health. Furthermore, among LMICs, the principal training systems differ in each country [47]. Therefore, the introduction of school health content appropriate to principal training processes in each country could be the key to the successful implementation of school health.

### Limitations

In the process of data collection from the FGDs, we invited participants from two leading ministries, the health and education departments. However, we could only interview participants from the health department of the school health supervisory board even though it consists of several departments. This might have negatively affected the comprehensive understanding of phenomena in this study because we could only obtain opinions from the health department in regard to cooperation between schools and relevant departments on the implementation of school health. Especially, the education department is in charge of pre-training and in-service training for teachers, which may have impacted the understanding of human resource development in this study. Further, the data from the FGDs conducted in schools may have been biased because the number of participants varied from school to school. In some schools, parent representatives or school board committee members were absent from FGDs, but we were able to conduct the FGDs in more than half of the schools with both parents and school board committee members present. Thus, we might have been able to invite as nearly diverse a group of members as possible to this study.

## Conclusion

In this study, themes were identified and generated for leadership practices related to the implementation of school health. It was found that schools with principals who demonstrated leadership in terms of the five elements of leadership shown in the Canadian study were found to promote school health. Additionally, the theme of “have professional educators’ beliefs and religious beliefs and Indonesia’s morals” was determined to be a novel theme compared to those in previous studies. The results of this study suggested that principals performed leadership practices while considering various religious diversity settings, collaborating with multiple sectors, and providing capacity building opportunities for the implementation of school health within schools in Mataram City, Indonesia. In several countries, education and religion are linked, and religious education cannot be ignored in the curriculum, especially in many countries where Islam is the state religion. It would be effective for principals to reflect their own religious values in their leadership in not only Indonesia, but also other countries, based on the multi-ethnic and multi-religious approach promoted in Indonesian civic education. Therefore, providing principals with training on school health policy might contribute to the implementation of school health. Furthermore, capacity building for principals in regard to school health might be applicable in other LMICs as it is assumed that the situation of school health in other LMICs is similar to that in Indonesia.

## Data Availability

Not applicable.

## Abbreviations

BOS: Bantuan Operasional Sekolah
FGDs: Focus group discussions
HPS: Health-promoting school
IDIs: In-depth interviews
LMICs: Low- to middle-income countries
UKS: Usaha Kesehatan Sekolah
UNESCO: United Nations Education, Scientific and Cultural Organization
WHO: World Health Organization
